# Supplementary material for: IL-33 Reduces Saturated Fatty Acid Accumulation in Mouse Atherosclerotic Foci
Source: Nutrients. 2024 Apr 17;16(8):1195. doi: 10.3390/nu16081195 (PMC11054828; doi:10.3390/nu16081195)
Supplement: Supplementary file 1 [file nutrients-16-01195-s001.zip › nutrients-2930866-supplementary.pdf]

A

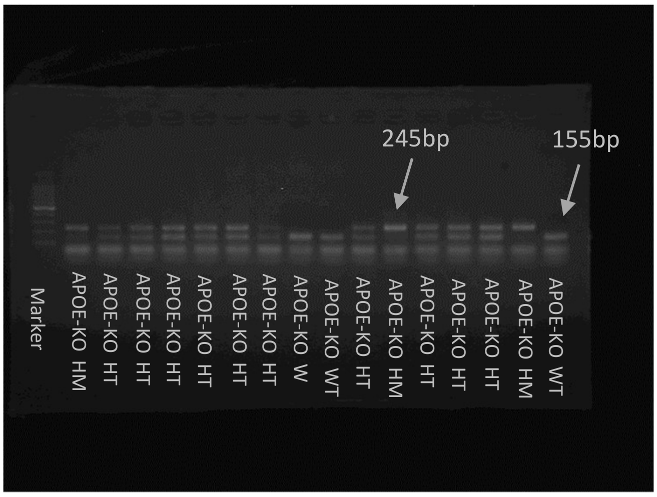

B

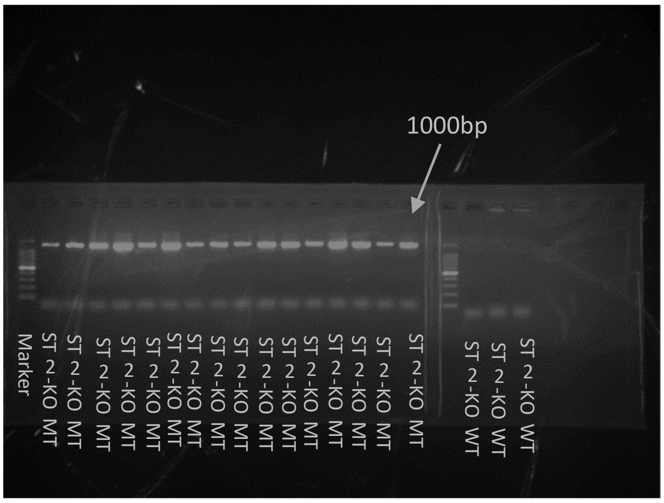

C

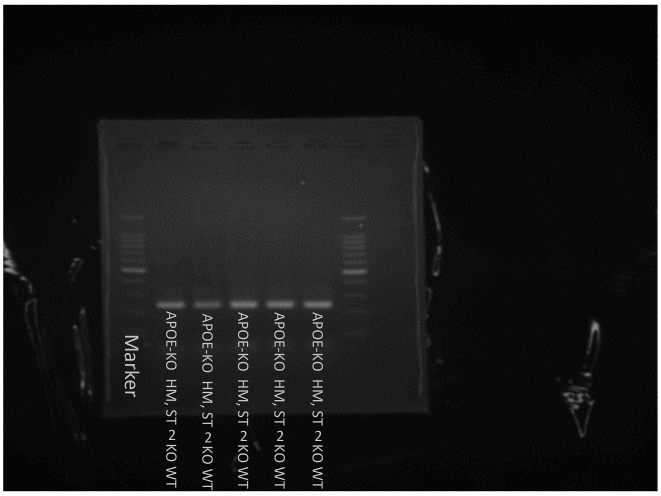

Supplementary Figure S1. Result of Genotyping the mouse. These shows the distribution of genotypes of specific genes in mouse (A)ApoEKO,(B) ST2KO,and(C) ApoEST2DKO. For genotyping mice, the tails of mice were used as samples. Samples were digested at 95° C for 5 min or 10 min.

(A) A band at 155bp is wild type, a band at 245bp is Homo type, and a double band at 155bp and 245bp is Hetero type.(B) The mutant type has a band around 1000 bp, while the wild type has no band. (C) The band around 245bp and the absence of a band around 1000 bp indicate ApoEKO (HM) and ST2KO (WT).
